# Characterization of the blood and neutrophil‐specific microbiomes and exploration of potential bacterial biomarkers for sepsis in surgical patients

**DOI:** 10.1002/iid3.483

**Published:** 2021-07-20

**Authors:** Chenyang Wang, Qiurong Li, Chun Tang, Xiaofan Zhao, Qin He, Xingming Tang, Jianan Ren

**Affiliations:** ^1^ Research Institute of General Surgery, Jinling Hospital Medical School of Nanjing University Nanjing China

**Keywords:** blood microbiome, high‐throughput sequencing, neutrophil‐specific microbiome, sepsis, septic shock

## Abstract

**Introduction:**

Recent studies have demonstrated the presence of a circulating microbiome in the blood of healthy subjects and chronic inflammatory patients. However, our knowledge regarding the blood microbiome and its potential roles in surgical patients remains very limited. The objective of this study was to determine the blood microbial landscape in surgical patients and to explore its potential associations with postoperative sepsis.

**Materials and Methods:**

2825 patients who underwent surgical treatments were screened for enrollment and 204 cases were recruited in this study. The patients were sub‐grouped into noninfected, infected, sepsis, and septic shock according to postoperative clinical manifestations. A total of 222 blood samples were obtained for neutrophil isolation, DNA extraction and high‐throughput sequencing, quantitative proteomics analysis, and flow cytometric analyses.

**Results:**

Blood and neutrophils in surgical patients and healthy controls contained highly diverse microbiomes, mainly comprising Proteobacteria, Actinobacteria, Firmicutes and Bacteroidetes. The majority (80.7%–91.5%) of the microbiomes were composed of gut‐associated bacteria. The microbiomes in septic patients were significantly distinct from those of healthy controls, and marked differences in microbiome composition were observed between sepsis and septic shock groups. Several specific bacterial genera, including *Flavobacterium*, *Agrococcus*, *Polynucleobacter*, and *Acidovorax*, could distinguish patients with septic shock from those with sepsis, with higher area under curve values. Moreover, *Agrococcus*, *Polynucleobacter*, and *Acidovorax* were positively associated with the sequential (sepsis‐related) organ failure assessment scores and/or acute physiology and chronic health examination scores in septic shock patients. The proteins involved in bactericidal activities of neutrophils were downregulated in septic patients.

**Conclusions:**

We present evidence identifying significant changes of blood and neutrophil‐specific microbiomes across various stages of sepsis, which might be associated with the progression of sepsis after surgical treatments. Several certain bacterial genera in blood microbiome could have potential as microbial markers for early detection of sepsis.

## Graphical abstract

(1) This study characterized for the first time the compositional features of the blood and neutrophli‐specific microbiomes in surgical patients and associated the microbiome alterations with the progression of postoperative sepsis. (2) Several certain bacterial genera in blood microbiome could have potential as microbial markers for early detection of sepsis. (3) The functional abnormality of neutrophils might play potentially intrinsic effectors in remodeling blood microbiome toward a disease‐provoking state in sepsis.

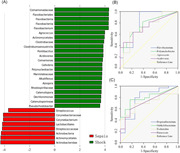

## 1 INTRODUCTION

Sepsis is a leading cause of death in ICU patients and is a major public health concern worldwide.^1^ Despite great advancements in new antimicrobial and intensive supportive care, the death rate of sepsis remains unacceptably high.^2^ Recently, sepsis has been defined as life‐threatening organ dysfunction caused by a dysregulated host response to infection.^3^ This definition highlights the primacy of a nonhomeostatic host response and potential lethality, yet a fundamental component for sepsis remains the presence of infection.^4^ Bacterial infection occurs frequently in surgical patients and is considered a key event in the development of postoperative sepsis. Early validation of infection is therefore of upmost importance, facilitating the determination that multiple organ dysfunction syndrome (MODS) is derived from a potential infection rather than other causes.

Over the last several decades, the disruption of intestinal barrier has been associated with sepsis and MODS.^5^, ^6^ Translocation of enteric organisms serves as the crucial step in the development of gut‐derived sepsis.^7^ Traditionally, the detection of translocating bacteria is dependent on microbiologic cultures from blood or relevant anatomic sites. However, negative cultures occur frequently in patients who are clinically identified as being septic.^1^, ^8^ The application of new technology, including real‐time polymerase chain reaction (PCR) and MALDI‐TOF MS, has improved the ability to detect pathogens^9^; however, our knowledge concerning the microbial landscape in the circulation of septic patients remains to be explored. Recent research with next‐generation sequencing has demonstrated the presence of a diverse bacterial microbiome in the blood of healthy subjects and patients with chronic diseases.^10^, ^11^, ^12^, ^13^, ^14^ This new paradigm raises questions concerning whether the blood harbors a rich microbiome in septic patients and is potentially associated with sepsis progression. Thereby, elucidation and characterization of the blood microbiome in septic patients is urgently needed, which might be helpful for achieving a better understanding of the microbiological nature of sepsis.

Neutrophils play a critical role in innate immunity and are essential for bacterial eradication and human polymicrobial sepsis survival.^15^ After internalization by neutrophils, the pathogens reside in the cytosol, shaping intracellular bacterial communities.^16^ Sepsis can induce persistent neutrophil dysfunction,^17^ likely causing an enrichment of intracellular pathogens and secondary infection.^18^ However, limited information regarding memberships of the intracellular community and its potential role in sepsis is available.

Using 16S rDNA‐based next‐generation sequencing, we characterized the compositional signatures of the bacterial microbiome presenting in peripheral blood and neutrophils of surgical patients during various stages of sepsis. We also sought to determine the possibility of gut‐associated bacteria as a major source of the blood microbiome and its contribution to the microbiome dysbiosis in sepsis. We further investigated the relationships of blood microbiome changes with immunological disorders, and the potential of some certain bacteria as microbial marker for the prediction of sepsis.

## 2 MATERIALS AND METHODS

### 2.1 Patients and sampling

**Figure 1:**
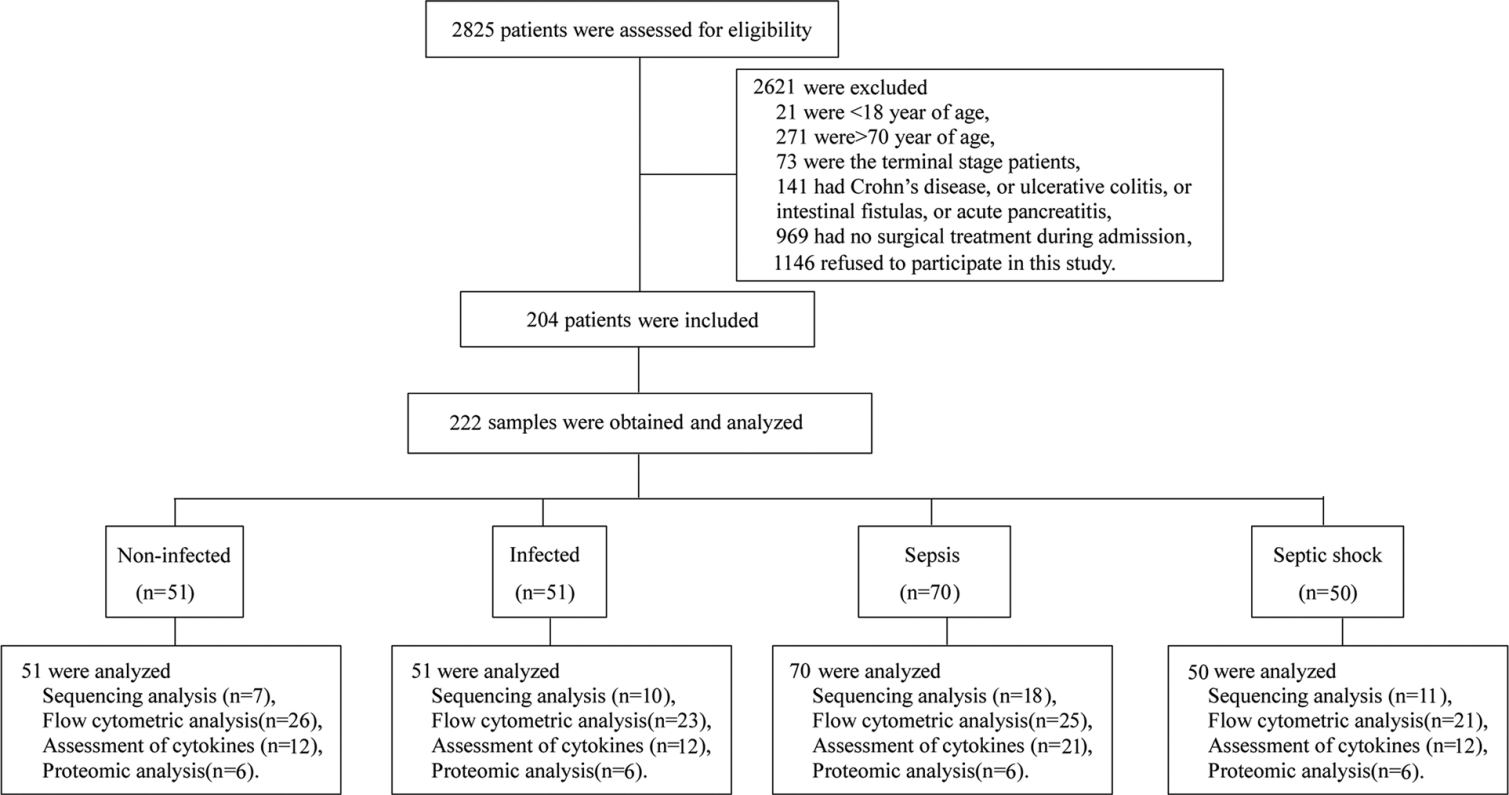
Study design and flow diagram. After a strict diagnosis and exclusion process, a total of 204 patients who underwent surgical treatments were included in this study. Two hundred and twenty‐two samples from noninfected (*n* = 51), infected (*n* = 51), sepsis (*n* = 70) and septic shock (*n* = 50) groups were further analyzed

To capture a broad range of stages of sepsis progression, 2825 patients who hospitalized in the Department of General Surgery at Jinling Hospital in China were prospectively assessed for possible enrollment. After careful evaluation, a total of 204 patients who underwent surgical treatments were recruited in this study. Based on postoperatively clinical manifestations, the patients were distributed into four groups: noninfected, infected, sepsis and septic shock. Sepsis and septic shock were identified with the most newly diagnostic criteria (Sepsis‐3).^3^ Infected patients were defined as having suspected or documented infections but no organ dysfunction, and noninfected cases showed neither infectious signs nor organ dysfunction. Peripheral blood samples were drawn under sterile conditions on the days when the infection, sepsis or septic shock was definitely diagnosed, and they were immediately delivered to our laboratory for further measurements. 46 of the patients enrolled in this study, including noninfected (*n* = 7), infected (*n* = 10), sepsis (*n* = 18) and septic shock (*n* = 11), were randomly chosen for high‐throughput sequencing analysis (Figure 1). Hematologic parameters, blood culturing outcomes and clinical characteristics of the patients are shown in Table 1. Healthy volunteers who had no signs of infection and no elevated serum CRP levels provided blood samples (*n* = 36) for further analyses.

**Table 1:** Demographics of study population for sequencing analysis

Variables			Noninfected	Infected	Sepsis	Septic shock
*N*			7	10	18	11
Age			49.6 ± 10.5	49.4 ± 17.9	54.2 ± 12.3	58.6 ± 13.4
Male (%)			2 (29)	5 (50)	11 (61)	11 (100)
ACHE II scores			3.7 ± 0.8	7.3 ± 3.6	15.3 ± 8.3	26.5 ± 12.5
SOFA scores			0.0 ± 0.0	1.7 ± 0.5	6.3 ± 4.2	14.2 ± 4.2
Lac (mmol/L)			N/A	2.30 ± 1.20	1.66 ± 0.70	5.25 ± 4.19
C‐reactive protein (mg/L)			54.8 ± 1.8	65.4 ± 38.5	105.3 ± 54.0	154.4 ± 50.2
Hematologic analysis	White blood cell count (×10^9^/L)		12.9 ± 3.5	15.5 ± 6.1	14.2 ± 8.4	20.6 ± 26.0
	Neutrophil percentage (%)		85.1 ± 8.6	86.9 ± 8.6	84.8 ± 11.3	92.9 ± 3.8
	Lymphocyte percentage (%)		10.1 ± 6.9	6.4 ± 4.4	7.9 ± 7.4	3.8 ± 3.5
	Hematocrit		0.38 ± 0.03	0.34 ± 0.05	0.31 ± 0.04	0.29 ± 0.06
	Platelet (×10^9^/L)		221.5 ± 50.6	317.3 ± 158.8	164.4 ± 102.7	93.1 ± 61.4
Liver function	Total protein (g/L)		61.6 ± 6.9	52.7 ± 8.3	53.9 ± 9.0	46.3 ± 6.2
	Albumin (g/L)		39.5 ± 2.2	31.4 ± 6.5	35.2 ± 4.4	32.6 ± 5.1
	Total bilirubin (μmol/L)		19.8 ± 33.9	13.9 ± 5.9	37.4 ± 53.8	54.5 ± 33.4
	Direct bilirubin (μmol/L)		14.0 ± 27.8	7.1 ± 4.3	26.0 ± 43.7	38.0 ± 31.0
	Indirect bilirubin (μmol/L)		5.9 ± 6.2	6.9 ± 4.5	10.4 ± 11.1	12.7 ± 7.1
Renal function	Creatinine (μmol/L)		52.6 ± 11.1	45.8 ± 18.7	91.9 ± 67.3	125.2 ± 73.6
	Urea *N* (mmol/L)		4.9 ± 1.9	3.7 ± 2.1	10.3 ± 6.2	9.2 ± 4.3
	Uric acid (μmol/L)		185.7 ± 62.7	130.0 ± 100.0	167.6 ± 111.3	178.1 ± 114.7
Blood coagulation	Prothrombin time (s)		11.4 ± 0.7	13.3 ± 1.2	14.7 ± 2.0	17.8 ± 4.7
	Partial thromboplastin time (s)		25.3 ± 3.8	44.2 ± 37.1	53.8 ± 33.7	66.0 ± 35.9
	International normalized ratio		1.0 ± 0.1	1.2 ± 0.1	1.3 ± 0.2	1.5 ± 0.4
	Fibrinogen (mg/dl)		3.0 ± 0.6	3.4 ± 1.1	3.2 ± 0.9	3.4 ± 1.2
Infection	Microbiologically confirmed (%)		0.0	10.0	44.4	45.5
	Clinically proven or suspected (%)		0.0	100.0	100.0	100.0
Blood culture	Gram^+^	*Staphylococcus* species (%)	0.0	0.0	11.1	0.0
	Gram^‐^	*Escherichia coli* (%)	0.0	0.0	0.0	18.2
		*Klebsiella* species (%)	0.0	10.0	11.1	18.2
		*Pseudomonas* species (%)	0.0	0.0	16.7	9.1
		*Enterobacter* species (%)	0.0	0.0	5.6	0.0
		Other (%)	0.0	0.0	11.1	9.1

### 2.2 DNA extraction, PCR, and 16S rDNA sequencing

Extraction of DNA from whole blood or isolated neutrophils was conducted in the biosafety cabinet (SterilGARD Ⅲ, The Baker Company) using the QIAamp DNA Mini Kit (Qiagen, Valencia, CA). The V3 region of the 16S rDNA was amplified with the universal primer set (357f/518r).^19^ Real‐time quantitative PCRs were firstly performed to validate the absence of bacterial contaminants from reagents and consumables (see Figure S1). Subsequently, an aliquot of DNA (100 ng) from each of samples was served as templates for PCR amplifications as we described previously.^20^ The amplicons were used for construction of barcoded libraries, and then sequenced using the Ion Torrent PGM system (Life Technologies) according to the manufacturer's instruction. The sequencing data were filtered, processed, and aligned taxonomically.^21^, ^22^ The α‐diversity of the microbiome, representing by species richness and phylogenetic diversity, was expressed by OTU numbers and Shannon diversity indices at the same sequencing depth, respectively. Heatmaps and principal coordinates analysis (PCoA) were performed using the R package (http://www.R-project.org/). To effectively detect differentially abundant features in the blood microbiome, the linear discriminant analysis effect size (LEfSe) algorithm among groups was conducted using the output matrix containing the relative abundance of OTUs per sample with an alpha cutoff of 0.05 and an effect size cutoff of 2.0 (http://huttenhower.sph.harvard.edu/lefse/).

### 2.3 Quantitative proteomics analysis

The proteins extracted from neutrophils were digested and labeled with 6‐plex iTRAQ reagents containing stable‐isotopes (Applied Biosystems) as we described previously.^20^ The labeled peptides were pooled, eluted and resolvedusing Ultremex SCX column (Phenomenex). The eluted fractions were desalted using a Strata X C18 column (Phenomenex). Subsequently, the peptides were subjected to nanoelectrospray ionization followed by tandem mass spectrometry (MS/MS) in a LTQ‐Orbitrap (Thermo Fisher Scientific) with a NanoACQUITY UPLC system. The resulting MS/MS spectra were searched using Maxquant (version 1.2.2.5) for validation of peptides and proteins.^23^ Gene Ontology (GO) functional annotation was carried out using Blast2GO software.^24^

### 2.4 Flow cytometry

Peripheral blood was freshly collected for flow cytometry analyses. Apoptosis of lymphocytes and neutrophils, T lymphocyte subpopulation, T helper (Th) cell subset, the expressions of HLA‐DR on monocytes, and the expressions of chemokine CXCR2 on neutrophils were measured. All antibodies and commercial kits were purchased from BD Biosciences. Stained cells were run on a FACSCanto II flow cytometer (BD Biosciences), and the resulting data were analyzed with FlowJo software (Tree Star Inc.).

### 2.5 Enzyme‐linked immunosorbent assay

Serum samples were collected and stored at −80°C for further analysis. Serum levels of cytokines, including tumor necrosis factor‐α (TNF‐α), interferon‐γ (IFN‐γ), interleukin‐1β (IL‐1β), IL‐2, IL‐6, IL‐10 and IL‐17, were determined using an enzyme‐linked immunosorbent assay (ELISA) kit (R&D Systems) according to the manufacturer's procedures.

### 2.6 Statistical analysis

Quantitative data are presented as the means ± standard deviation (*SD*). Statistical analysis was conducted by one‐way analysis of variance (ANOVA) with post hoc test (least significant difference) using SPSS software (version 12.0). A *p* value of less than .05 represented significant difference between groups. Correlations between two variances were estimated using linear regression analysis with a Pearson's test. Receiver operating characteristic (ROC) curves were used to determine the bacterial genera that might predict the progression of sepsis in surgical patients.

### 2.7 Ethics statement

This study was approved by the Institutional Ethical Committee of Jinling Hospital (2018JLHLS‐132) and was performed in accordance with the Declaration of Helsinki and Good Clinical Practice Guidelines. Written informed consent for study participation was obtained from all participants or legally authorized representative.

## 3 RESULTS

### 3.1 Characterization of the blood microbiome in surgical patients

**Figure 2:**
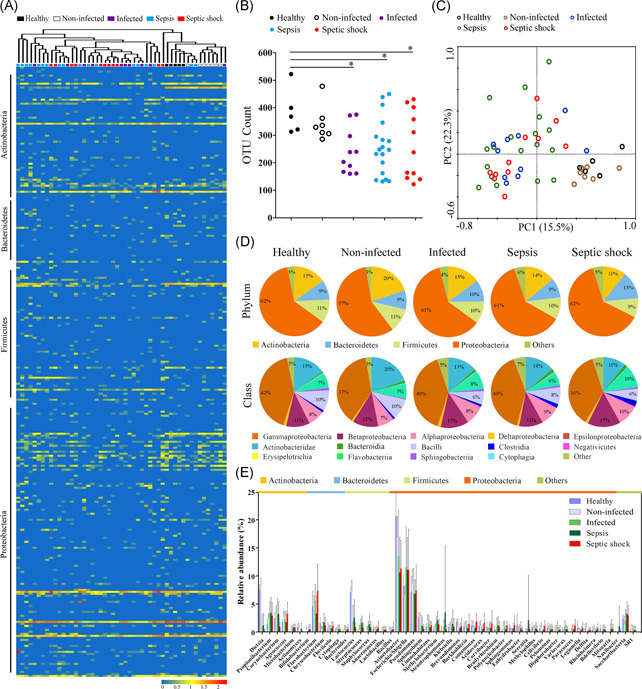
Composition and diversity of blood bacterial microbiome in surgical patients across various stages of sepsis. (A) The relative abundance of the bacterial genera identified by taxonomic classification, as revealed by a heatmap graph. The clustering relationships across the blood samples are shown in the upper panel. (B) Comparative analysis of the species richness of the blood microbiomes among groups. The species richness is expressed as the counts of the observed OTUs at the same sequencing depth. *p < .05 versus healthy subjects. (C) Principal coordinate analysis (PCoA) plot of weighted UniFrac distances between the blood samples from five groups. (D) Determination of the predominant bacterial composition in the blood microbiome at the phylum and class levels. (E) Shifts in the relative abundance of the top 50 most abundant bacterial genera among groups

To profile the microbial landscape in systemic circulation, we sequenced the 16S rDNA recovered from the peripheral blood of 46 patients and 5 healthy subjects. We detected that a diverse bacterial microbiome was present in the blood of patients and healthy subjects, which was mainly composed of the phyla Proteobacteria, Actinobacteria, Bacteroidetes and Firmicutes (Figure 2A). To characterize differences in the microbiome composition between healthy and patient groups, we analyzed the α‐diversity, as assessed by the species richness (OTU numbers at the same sequencing depth) and phylogenetic diversity (Shannon indices). The blood microbiomes of septic patients, including sepsis and septic shock, had a lower community richness (Figure 2B), whereas no significant difference in phylogenetic diversity versus healthy individual was observed (data not shown). The microbiome composition in septic and infected populations appeared different from healthy subjects, as evidenced by a clear separation between the communities along the first and second principal coordinate (Figure 2C). However, noninfected and healthy groups clustered together, showing high community similarity. Analyses at different taxonomic levels indicated that the blood microbiome was dramatically altered in septic patients, especially in septic shock. At the phylum level, the most significant shifts were observed in patients with septic shock, characterized by an increase in Bacteroidetes and a reduction in Actinobacteria versus healthy subjects (*p* < .05) (Figure 2D). The increase in Bacteroidetes was mainly due to expansion of the classes Flavobacteriia and Bacteroidia (*p* < .05), and the reduction of Actinobacteria was largely caused by Actinobacteridae (*p* < .05) (Figure 2D). Betaproteobacteria and Clostridia were also significantly more abundant in septic shock, whereas Gammaproteobacteria and Bacilli showed a reduced presence compared with the healthy group (*p* < .05). Class‐level differences between sepsis and healthy groups were relatively lower, mainly derived from increases in Clostridia and Bacteroidia (*p* < .05). Genus‐level analyses revealed that specific bacterial phylotypes contributed to alterations of blood microbiomes in septic patients (Figure 2E). The genera *Lactococcus*, *Dietzia*, and *Sphingobium* were markedly reduced in septic patients (*p* < .05) (Figure S2), while *Escherichia/Shigella*, *Propionibacterium*, *Methylobacterium*, and *Bradyrhizobium* were increased versus healthy subjects (*p* < .05) (Figure S3). The genera *Staphylococcus*, *Serratia*, *Paracoccus*, and *Mitsuaria*, which were absent in the healthy group, were prevalent in septic patients (*p* < .05) (Figure S4). In addition, the genera *Flavobacterium*, *Agrococcus*, *Polynucleobacter*, S*phingomonas*, and *Curvibacter* exhibited a profound expansion in septic shock, but not in sepsis (*p* < .05) (Figure S5). The disturbance of blood microbiomes seemed to be aggravated with the progression of sepsis towards septic shock.

### 3.2 Shifts of the neutrophil‐specific microbiome in surgical patients

**Figure 3:**
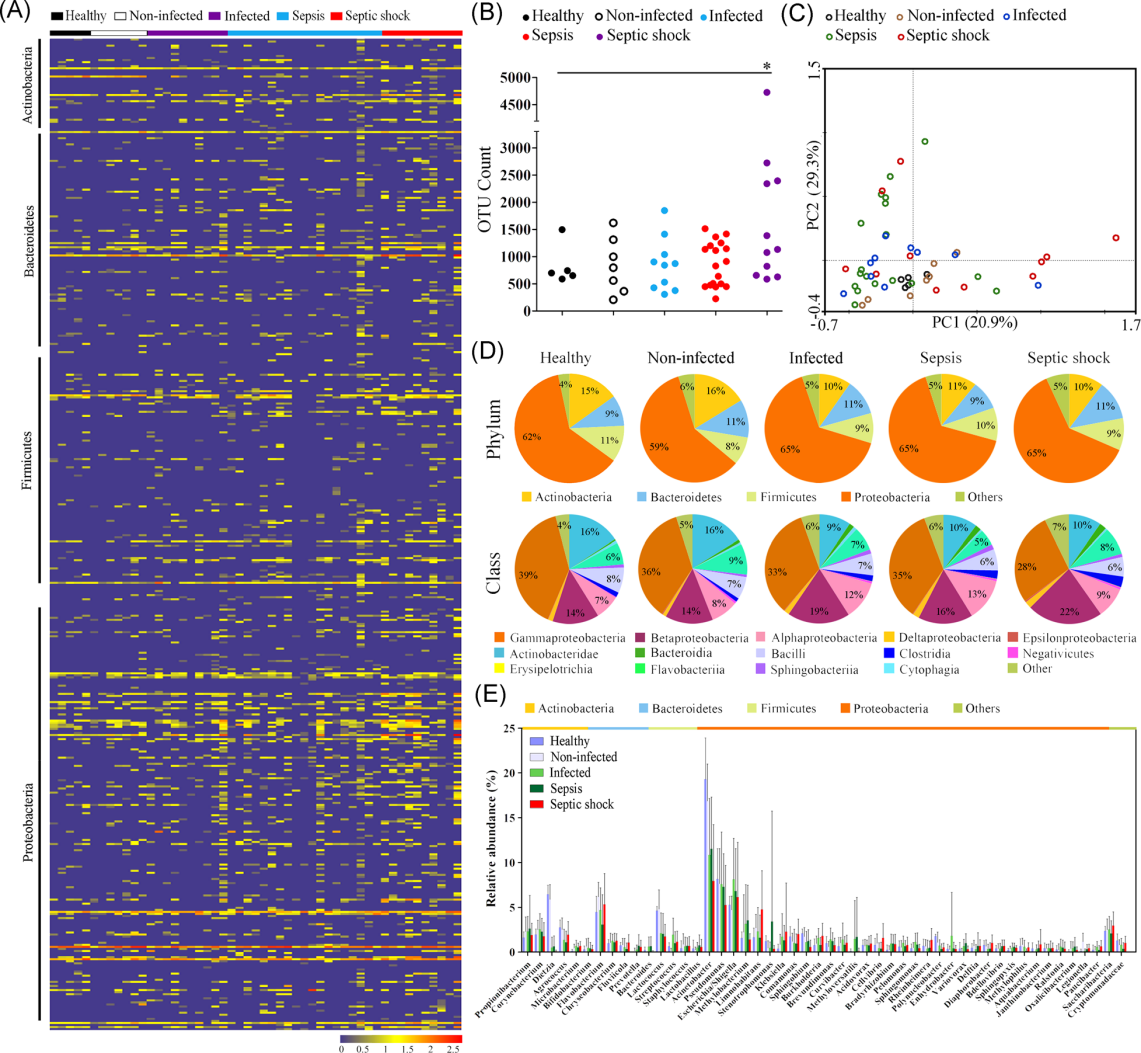
Shifts of neutrophil‐specific bacterial microbiome in neutrophils of surgical patients across various stages of sepsis. (A) Heatmap displaying alterations in the relative abundance of the intracellular bacterial genera. (B) Comparative analysis of the species richness of the blood microbiomes among groups. The species richness is expressed as the counts of the observed OTUs at the same sequencing depth. *p < .05 versus healthy subjects. (C) PCoA plot of weighted UniFrac distances between samples from the five groups. (D) Determination of the predominant bacterial composition in the intracellular communities at the phylum and class levels. (E) Variations in the relative abundance of the top 50 most abundant bacterial genera among groups

Next, we isolated neutrophils and profiled its intracellular bacterial communities, also termed the neutrophil‐specific microbiome (Figure 3A). Interestingly, the neutrophil‐specific microbiome composition resembled that of the blood microbiome within each group (Figure S6). The structure and composition of neutrophil‐specific microbiome were also altered in septic patients. While the phylogenetic diversity showed no significant difference between septic and healthy groups, the species richness significantly increased in septic shock (*p* < .05) (Figure 3B). The majority of septic samples, especially septic shock, were clustered separately from the healthy controls (Figure 3C), indicating significant differences in the neutrophil‐specific microbiome composition between groups. Compared with healthy individuals, the microbiomes in septic patients were characterized by a reduced proportion in Actinobacteria and increased level in Proteobacteria (*p* < .05) (Figure 3D). At the class level, shifts of neutrophil‐specific microbiome in septic patients were mainly attributed to the reduction of Actinobacteridae and increase in betaproteobacteria and alphaproteobacteria (*p* < .05) (Figure 3D). We also observed a dramatic reduction in the proportion of gamaproteobacteria in septic patients (*p* < .05). Of the top 50 most abundant genera, the relative proportions of *Lactococcus*, *Dietzia*, *Sphingobium*, and *Polynucleobacter* markedly declined in septic patients, especially in septic shock (*p* < .05) (Figures 3E and S7). In contrast, *Escherichia/Shigella*, *Klebsiella* and *Bradyrhizobium* were more abundant in septic groups than in healthy controls (*p* < .05) (Figure S8). *Paracoccus*, *Limnohabitans Burkholderia*, and *Flavobacterium* were significantly increased in septic shock patients (*p* < .05) (Figure S9). In total, the neutrophil‐specific microbiome was severely altered in septic patients, in particular septic shock, similar to the observations from blood microbiomes.

### 3.3 Potential source of blood and neutrophil‐specific microbiomes

To identify the possible source of blood and neutrophil‐specific microbiomes, we compared our sequences to the 16S rRNA gene data set from different anatomical sites of healthy individuals in the Human Microbiome Project.^25^ We found that the gut‐associated bacteria contributed the majority (range 80.7%–91.5%) of blood and neutrophil‐specific microbiomes, representing a far higher amount than the other sites (Figure S10). We then investigated the composition of the gut‐associated bacterial community to unravel its relationship with microbiome‐wide dysbiosis in septic patients. Remarkably, variation trends of gut‐associated microbiomes in septic patients, including species richness, phylum‐ and class‐level composition (Figure S10), were consistent with those of whole bacterial communities in peripheral blood (Figure 2B,D) and neutrophils (Figure 3B,D). The data suggested that gut‐associated bacteria were a major source of blood and neutrophil‐specific microbiomes and that its changes were closely involved in microbiome‐wide dysbiosis in septic patients.

### 3.4 Association of immune dysfunction with microbiome dysbiosis

**Figure 4:**
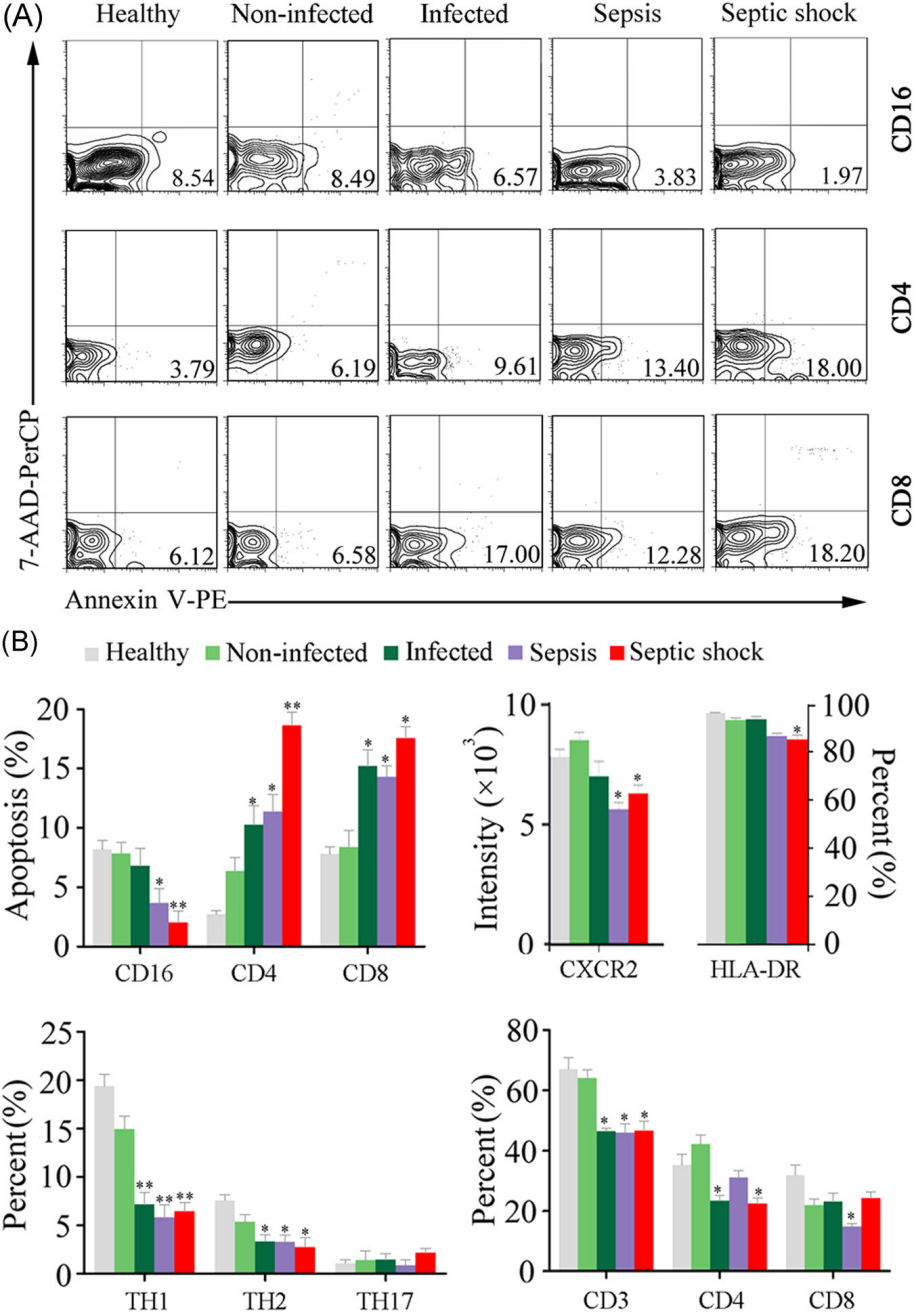
Variations of the peripheral immune cells in septic patients. (A) Representative histograms illustrating the cell apoptosis of the neutrophils and T lymphocytes. (B) Statistical analysis of flow cytometry results showing the apoptosis and subset changes of immune cells. *p < .05; **p < .01; versus healthy subjects

To explore associations between systemic immunity and blood microbiome memberships, we characterized the major members of innate and adaptive immune cells in blood. Septic patients showed typical immune alterations in innate cell types, characterized by increased neutrophil counts and delayed apoptosis (*p* < .05) (Figure 4A,B).^17^, ^26^ We also observed a marked decline in expression of CXCR2 on neutrophils of septic patients (*p* < .05), indicating the functional deficits in cell migration.^27^ The proportion of HLA‐DR expressing monocytes was also reduced in septic patients, especially in septic shock. By contrast, the apoptosis of CD4^+^ and CD8^+^ T cells significantly increased in septic patients (*p* < .05) (Figure 4B). The proportions of Th1 and Th2 cells strikingly reduced in septic patients (*p* < .05). Intriguingly, the peripheral immunological changes, including the percentages of apoptotic cells and lymphocyte subsets together with serum cytokine levels, were closely related to changes in blood and neutrophil‐specific microbiomes in septic patients (Figure S11).

### 3.5 Changes of circulating neutrophil functionality by quantitatively proteomic analysis

**Figure 5:**
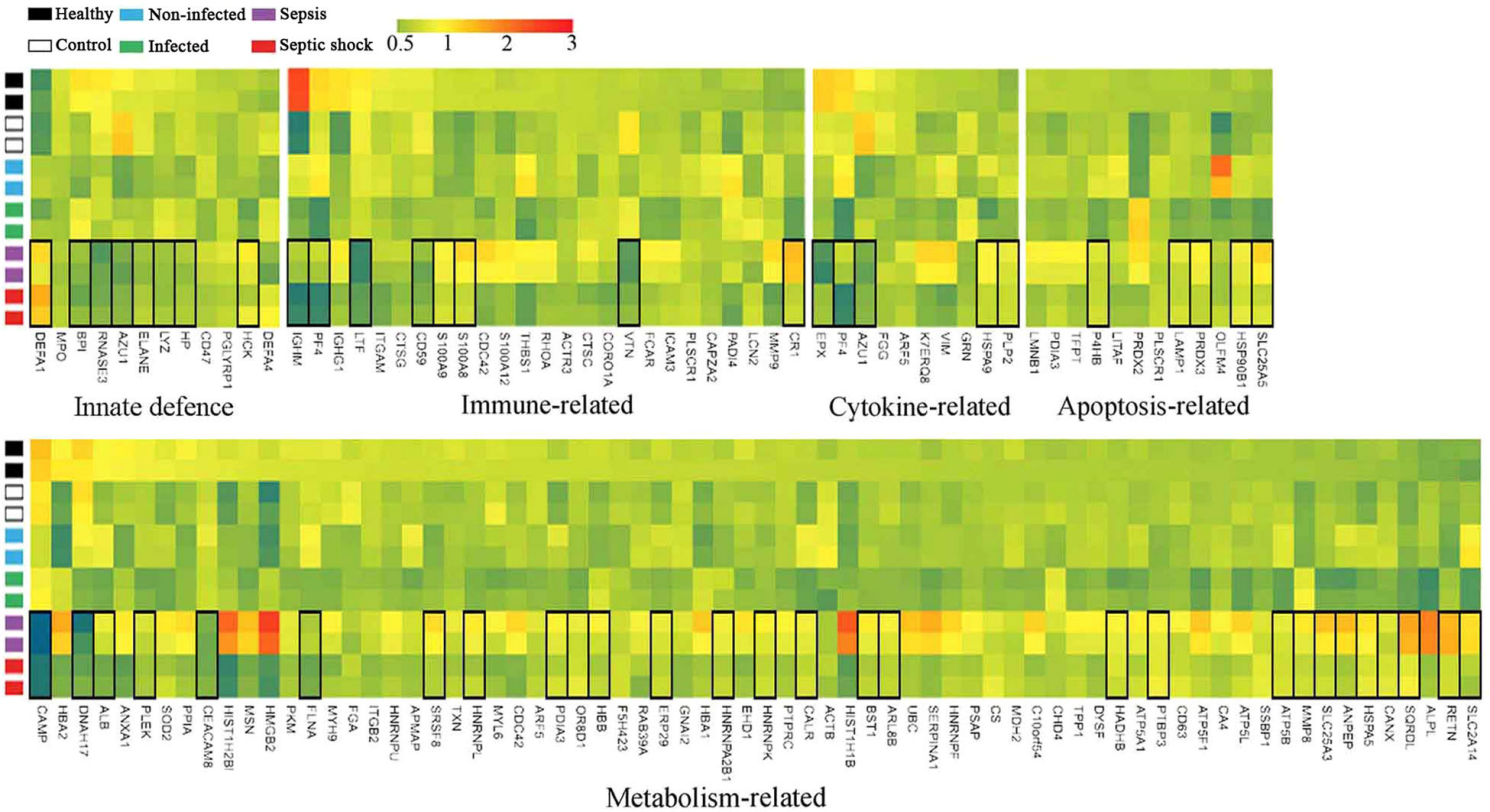
Proteomics profiling of circulating neutrophils by quantitatively iTRAQ analysis. Heatmap showing the changes of the relative abundance of expressed‐differentially proteins in neutrophils

To connect the functionality of neutrophils to blood microbiome‐wide dysbiosis, we performed quantitatively proteomic analysis on neutrophils derived from septic patients. A total of 129 proteins were defined as differentially expressed, clustered into five functional categories: innate immune defense, immune regulation, cell apoptosis, cytokine release and metabolic activity (Figure 5). The proteins involved in bactericidal activities of neutrophils,^28^ such as bactericidal permeability‐increasing protein (BPI), elastase (ELANE), cathepsin G (CTSG), azurocidin (AZU4), cathelicidin antimicrobial peptide (CAMP), and myeloperoxidase (MPO) were downregulated in septic patients. Intriguingly, expression of these proteins was abnormally lowered in septic shock. Some of immune regulation‐associated proteins, including integrin alpha‐M (ITGAM), IgA Fc receptor (FCAR), and lactotransferrin (LTF) were significantly downregulated in septic patients (Figure 5). Matrix metallopeptidase 9 (MMP9), a protein prompting leukocyte trans‐endothelial migration, was pronouncedly downregulated in septic shock patients, suggesting impaired migration activity of neutrophils.^29^ Some apoptosis‐related proteins were markedly unregulated in sepsis and septic shock. In addition, an over‐representation of the functional proteins involved in metabolism was observed in septic patients. The global variations of the proteomic profiles provided evidence that the function of neutrophils was collapsed in septic patients, particularly in septic shock.

### 3.6 Blood microbial markers for predicting the progression of sepsis

**Figure 6:**
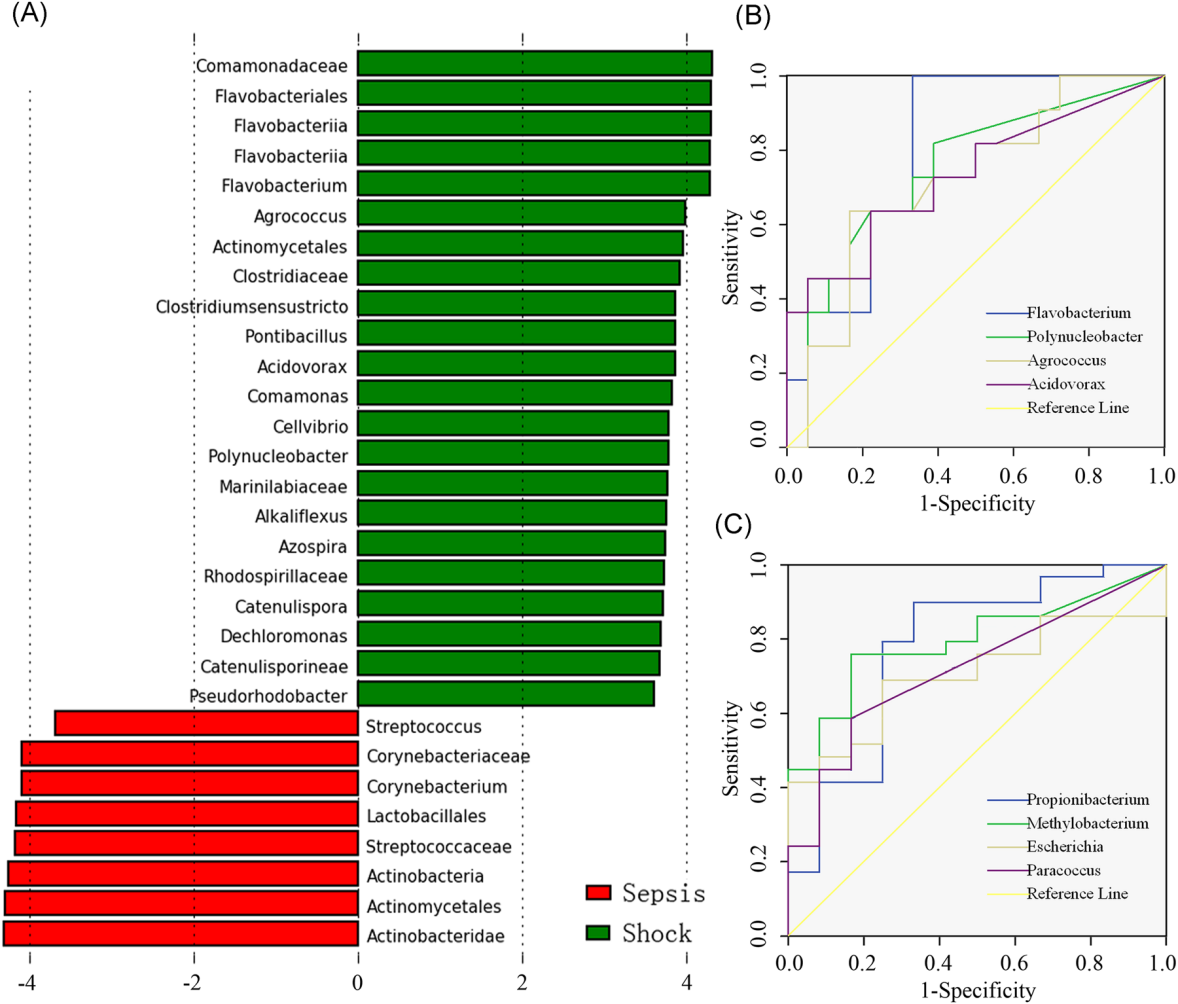
Variations of blood bacterial microbiome in septic shock patients. (A) Significantly discriminative taxa between the septic shock patients and sepsis cases were determined by linear discriminant analysis effect size (LEfSe) analysis. The green bar chart represents the bacteria taxa enriched in blood samples of septic shock patients, and the red bar chart represents the sepsis individuals. (B) Receiver operating characteristic (ROC) curves for prediction values of some specific bacterial genera. ROC curves of discriminating patients with septic shock from sepsis patients (95% confidence interval). (C) ROC curves of discriminating septic patients (including sepsis and septic shock) from noninfected controls

**Figure 7:**
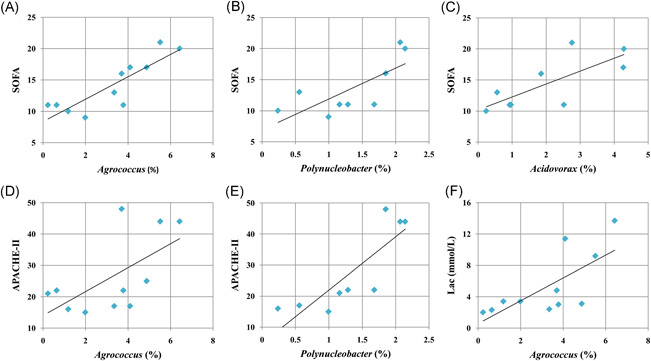
Associations between specific bacterial clades in blood and clinical signatures of patients with septic shock. (A–C) The relative proportions (%) of the genera *Agrococcus*, *Polynucleobacter*, and *Acidovorax* were positively associated with the Sequential (Sepsis‐related) Organ Failure Assessment (SOFA) scores in patients with septic shock. (D, E) The genera *Agrococcus* and *Polynucleobacter* correlated positively to the acute physiology and chronic health examination (APACHE‐II) scores in septic shock patients. (F) *Agrococcus* was positively related to levels of serum lactate in septic shock

Given the connection between blood microbiome and clinical signatures, we generated receiver operating characteristic (ROC) curves to search for some bacterial genera that might predict the progression of sepsis. According to LEfSe analyses, 22 bacterial genera, including *Flavobacterium*, *Agrococcus*, *Polynucleobacter*, *Acidovorax*, *Clostridium sensustricto*, and *Comamonas*, among others, were significantly enriched in patients with septic shock compared with sepsis patients (Figure 6A). Four bacterial genera among them, *Flavobacterium*, *Agrococcus*, *Polynucleobacter* and *Acidovorax*, could distinguish patients with septic shock from patients with sepsis, with area under curve (AUC) values of 0.808, 0.745, 0.72, and 0.732 (Figure 6B). Four bacterial genera in the blood, including *Propionibacterium*, *Methylobacterium*, *Escherichia/Shigella*, and *Paracoccus* discriminated septic patients (including sepsis and septic shock) from controls, with AUC values of 0.776, 0.796, 0.704, and 0.718, respectively (Figure 6C). Specifically, the genera *Agrococcus*, *Polynucleobacter* and *Acidovorax* were positively associated with the sequential (sepsis‐related) organ failure assessment (SOFA) scores in patients with septic shock (*p* = .0007, .0212 and .0185, respectively) (Figure 7A–C). *Agrococcus* and *Polynucleobacter* correlated positively to the acute physiology and chronic health examination (APACHE‐II) scores in septic shock patients (*p* = .0443 and .0044, respectively) (Figure 7D,E). *Agrococcus* was positively related to serum lactate levels in septic shock (*p* = .0132) (Figure 7F).

## 4 DISCUSSION

In this study, we present emerging evidence for the presence of blood and neutrophil‐specific microbiomes in surgical patients and healthy subjects. We show that the microbiome composition is dramatically altered in sepsis, and dysbiotic shifts appear aggravated with the progression of sepsis towards septic shock. Further, the microbiome dysbiosis is closely linked to immune dysfunction and an elevated inflammatory response in septic patients. More importantly, we identify unique compositional signatures of septic microbiomes, certain members of which have the potential as microbial markers for the predication of sepsis, especially septic shock.

Recently, several studies have demonstrated that human blood contains a diverse bacterial microbiome, which might be involved in the development of some chronic diseases.^11^, ^12^, ^13^, ^14^ However, whether a diverse microbiome presents in the peripheral circulation of postoperative patients with infection is still an unanswered question. Using 16S rDNA‐based denaturing gradient gel electrophoresis techniques, multiple bacterial species were observed in the peripheral blood in severe acute pancreatitis patients with bacteraemia,^30^ but without next‐generation sequencing analyzing, it is impossible to characterize the features of the blood microbial landscape in the surgical patients and its involvement in the progression of sepsis. In the present study, we conducted 16S rDNA sequencing to profile the bacterial microbiome in the blood of surgical patients. We showed that the blood in surgical patients is replete with a diverse microbiome, dominated by Proteobacteria, Actinobacteria, Bacteroidetes and Firmicutes, consistent with previous observations in chronic diseases.^14^ The blood microbiome in the patients with postoperative sepsis appears dysbiotic, and the shifts are aggravated with the development of sepsis towards septic shock. Dysbiosis of the blood microbiome has been reported as an independent risk factor for cardiovascular disease, suggesting its potentially pathological role in the development of chronic inflammation.^11^, ^12^, ^13^ Importantly, we also showed that alterations of the microbiome memberships are closely related to clinical manifestations of patients, especially the disease severity and immunological disorders. As such, it is possibly speculated that the shifts of blood microbiome composition might represent a disease‐provoking state, which likely contributes to the progression of sepsis.

The dysbiosis of gut microbiota and bacterial translocation are frequently seen in critically ill patients. A great amount of evidence has indicated that the translocation of bacteria and their products across intestinal barrier can drive the pathogenesis of sepsis.^21^, ^22^, ^23^, ^24^, ^25^, ^26^, ^27^, ^28^, ^29^, ^30^, ^31^, ^32^, ^33^, ^34^ Basing on blood culture studies, in the last several decades the concept of bacterial translocation is defined as translocation of one or several microorganisms and/or endotoxin from the gut.^35^, ^36^, ^37^, ^38^ Recent studies have demonstrated that the lung in patients with sepsis and acute respiratory distress syndrome contains a diverse microbiome, which is mainly composed of the bacterial microbiota from the gut, but not from the oral or upper respiratory tract.^39^ These findings prompted us to re‐consider the current opinion of bacterial translocation from intestinal tract to the peripheral blood. In this study, we show that the blood microbiome in the patients is mostly made up of gut‐derived organisms (range 80.7–91.5%), similar to the results from the lung microbiome.^39^ Our investigations also demonstrated a previously unrecognized complexity of the circulating microbiome in the surgical patients. In combination with the findings, the definition of bacterial translocation should be re‐defined as translocation of the bacterial microbiota from the gut.

In systemic circulation immune cells and invaded microbes are highly interactive, which is of special importance for maintaining a delicate balance between defences against infection and eliciting an excessive inflammatory response.^40^ In view of the central role of neutrophils in eradication of pathogens, we characterized the compositional feature of the intracellular bacterial communities in neutrophils of surgical patients and explored their potential role in the dysbiosis of the circulating microbiome in sepsis. We showed that the neutrophil‐specific microbiome was significantly altered in septic patients, consistent with the findings from the blood microbiome. We also observed delayed neutrophil apoptosis in septic patients, likely leading to functional deficiencies in bacterial clearance.^17^ In addition, a significant increase in lymphocyte apoptosis was observed in septic groups, indicating the presence of an adoptive immune disorder. Persistent dysfunction in circulating neutrophils may cause a failure of bacterial eradication (especially for organisms that have invaded cells), leading to profound alterations in the neutrophil‐specific microbiome in septic patients. Ultimately, a dysregulated host response to invaded organisms may cause the progression of sepsis and even septic shock. It might be possible that the functional abnormality of neutrophils might play potentially intrinsic effectors in remodeling blood microbiome toward a disease‐provoking state in sepsis.

Sepsis is among the most frequent complications in surgical patients and is considered the primary cause of the mortality from infection, especially if not recognized and treated promptly.^3^ Unfortunately, it is particularly difficult to predict the presence of sepsis and MODS in patients, even for the experienced clinicians. Thereby, the development of new biomarkers is urgently needed for the prediction of sepsis in surgical patients. Recently, gut microbiome analyses have served as a tool for targeted noninvasive biomarkers for several chronic diseases and cancers.^41^, ^42^, ^43^, ^44^ However, it is unclear whether the circulating microbiome could be used to predict the presence of sepsis and organ dysfunction after surgery. In this study, we showed that four microorganisms presenting in the circulating microbiome, including *Propionibacterium*, *Methylobacterium*, *Escherichia/Shigella* and *Paracoccus*, could discriminate septic patients from noninfected controls, with higher AUC values. The data suggest the potential of such organisms as predictive indicators for postoperative sepsis. Septic shock is associated with greater mortality rates than sepsis alone, and it is also very difficult to predict the presence of septic shock from sepsis. Here, the bacterial genera *Flavobacterium*, *Agrococcus*, *Polynucleobacter* and *Acidovorax* displayed the potential to distinguish patients with septic shock from patients with sepsis. Moreover, *Agrococcus*, *Polynucleobacter* and *Acidovorax* were positively related to the SOFA scores in septic shock patients. Our findings presented herein are intriguing and provide a novel direction to search for more sensitive and specific biomarkers for the prediction of sepsis and organ dysfunction. In the future, a prospective study containing a larger cohort of surgical patients is required to validate the predictive value of these microbial markers. Overall, 16S rDNA‐based signatures of the circulating microbiome appear to be much more sensitive and could be developed as a useful laboratory tool for predicting sepsis and septic shock in surgical patients. Combining blood microbiome detection and clinical signs may allow the more precise prediction of sepsis and septic shock, likely leading to earlier interventions and improved clinical outcomes.

In the last century, the culture‐based method has been viewed as the golden standard for the identification of microbes. However, a large variety of studies have suggested that blood culture results may not necessarily reflect the true bacteriologic status in systemic circulation.^1^, ^8^ Culture‐independent techniques have shown great benefits for deeply dissecting the composition of bacterial flora, even for ultra‐low‐diversity communities.^45^, ^46^ Here, we sought to characterize associations between microbiologic culture results and microbiome profiles. Of the 29 septic patients, 13 were marked by at least one positive blood culture (Table 1). The pathogens isolated most frequently in the patients were *Staphylococcus aureus*, *Enterococcus*, *Escherichia coli*, *Klebsiella*, and *Pseudomonas*. Not surprisingly, these organisms presented as the predominant phenotypes in the blood microbiome of septic patients, but no significant relationships were discerned between a higher abundance and positive culture results. Through 16S rDNA‐based high‐throughput sequencing, hundreds of bacterial phenotypes can be detected in blood without a positive culture, indicating that the culture‐independent approach could be more useful to profile the microbial landscape in blood, likely leading to an improved understanding of the pathogenesis of sepsis. However, culture‐independent sequencing techniques also have limitations. It is difficult to determine whether the microbial DNA sequence presenting in the blood represents a “live” bacterial species, which is extremely important for precision medical treatment against microbial pathogens in clinical practice. Although the high sensitivity of DNA sequencing changes our general concept that blood is sterile, this is still a controversial field.^47^, ^48^ Actually, blood is not lacking of bacterial products except more bacteria during infection. It was generally believed that bacteria enter the circulation to induce sepsis. However, the bacteria entering circulation are normally not harmful unless some specific stains. Therefore, analyzing bacteria in blood is not that important except to identify antimicrobial resistance (AMR) fragments. Future studies should pay more attention to identifying harmful pathogens presenting in the blood.

Taken together, we have added considerable evidence that the blood contains a diverse bacterial microbiome in surgical patients. The blood microbiomes in surgical patients are dramatically altered across various stages of sepsis, and the shifts are aggravated with the progression of sepsis towards septic shock. Furthermore, alterations of the microbiome memberships are closely related to the organ dysfunction and illness severity in septic shock. Previous studies have shown robust associations of the blood microbiome with systemic inflammation in patients with chronic liver diseases.^49^, ^50^, ^51^ In combination with our current findings, it can be speculatively concluded that the dysbiosis of the circulating microbiome could be reasonably presumed to increase the risk of postoperatively adverse events, including infection, sepsis and septic shock. In addition, early assessment of the blood microbiome in surgical patients is critically needed, which may be used to predict the progression of postoperative sepsis. Even though sepsis does not require a diagnosis from blood microbiome analysis, these investigations could contribute to an increased awareness among clinicians that the alternations in the circulating microbiome might be associated with septic complications after surgical interventions. Nonetheless, this is a preliminary study containing smaller samples. Future studies with a larger cohort and substantial samples collected from each patient are warranted to validate the findings of this study and evaluate the net effect of the microbiome alternations on the progression and outcome of sepsis in surgical patients. It is also needed to offer compelling evidence for the true presence of gut‐blood microbiota translocation through metagenomic sequencing analyzing of paired stool and blood specimens in sepsis.

## AUTHOR CONTRIBUTIONS

All authors have contributed to the final version of the manuscript as follows. Study concept and design: Qiurong Li, Chenyang Wang, Jianan Ren. Acquisition, analysis, or interpretation of data: Chenyang Wang, Qiurong Li, Chun Tang, Xiaofan Zhao, Qin He, Xingming Tang. Drafting of the manuscript: Chenyang Wang, Qiurong Li. Statistical analyses: Chenyang Wang. Critical revision of the manuscript: Chenyang Wang, Qiurong Li. All authors read and approved the final manuscript.

## CONFLICT OF INTERESTS

The authors declare that there are no conflict of interests.

## Supporting information

Supplementary information.

## Data Availability

The data that support the findings of this study are available from the corresponding author upon reasonable request.
