# Supplementary material for: Characterization of the blood and neutrophil‐specific microbiomes and exploration of potential bacterial biomarkers for sepsis in surgical patients
Source: Immun Inflamm Dis. 2021 Jul 20;9(4):1343–57. doi: 10.1002/iid3.483 (PMC8589375; doi:10.1002/iid3.483)
Supplement: Supplementary file 1 — Supplementary information. [file IID3-9-1343-s001.pdf]

Supplementary Figures

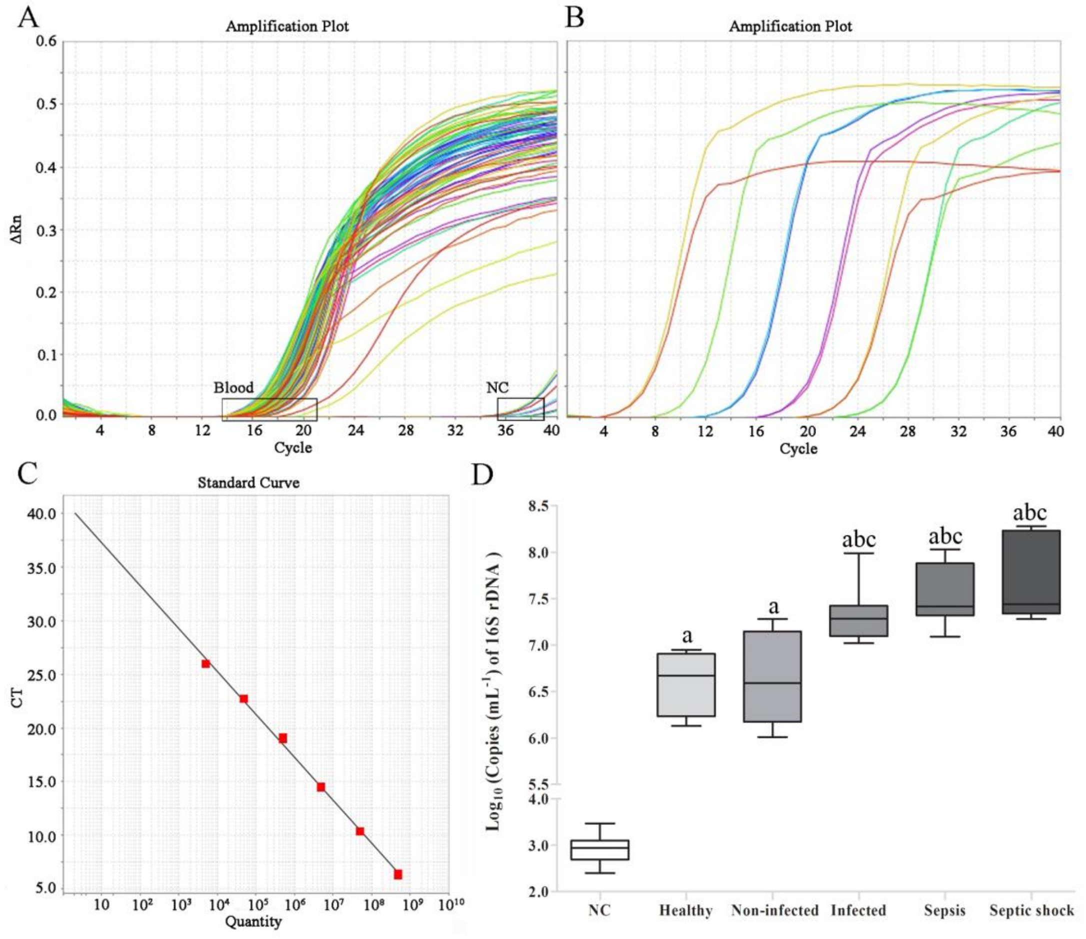

Figure S1. Determination of the bacterial 16S rDNA in blood samples by quantitative polymerase chain reaction (qPCR) assay. **A**, Amplification plots of qPCR obtained from blood samples and negative controls (NC). The assays were performed on the AB7500 real time PCR system (Life Technologies, CA). **B,** Amplification plots generating from a series of 10-fold dilutions from 5×10^3 to 5×10^8 of 16S rRNA gene copies per reaction. **C**, Standard curve generating from the standard dilutions. **D**, Comparison of the bacterial 16S rDNA concentrations in blood samples between patients and healthy controls (HC). The NC indicates the background signal from the reagents and consumables. a: $p<0.001$, *vs*. NC; b: $p<0.01$, *vs*. Healthy; c: $p<0.01$, *vs*. Non-infected.

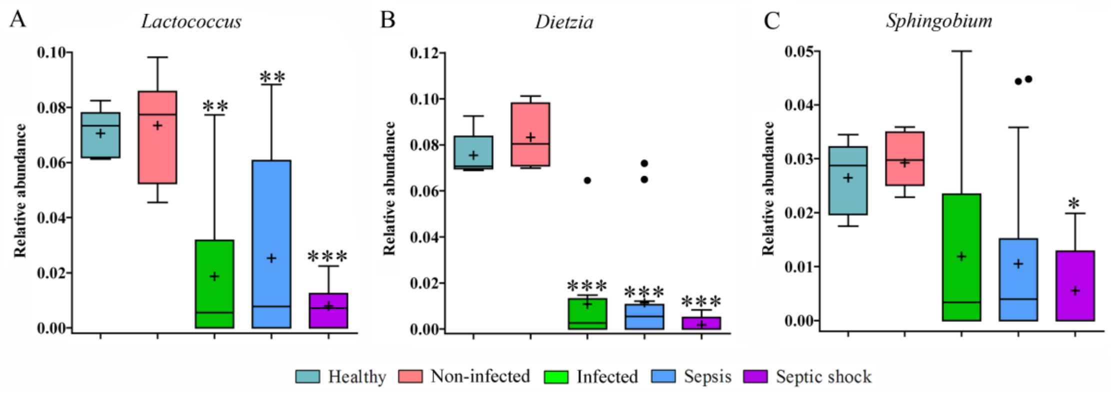

Figure S2. The blood bacterial genera showing significant reduction in their relative abundance in septic patients. * p<0.05, ** p<0.01, *** p<0.001,*vs*. Healthy.

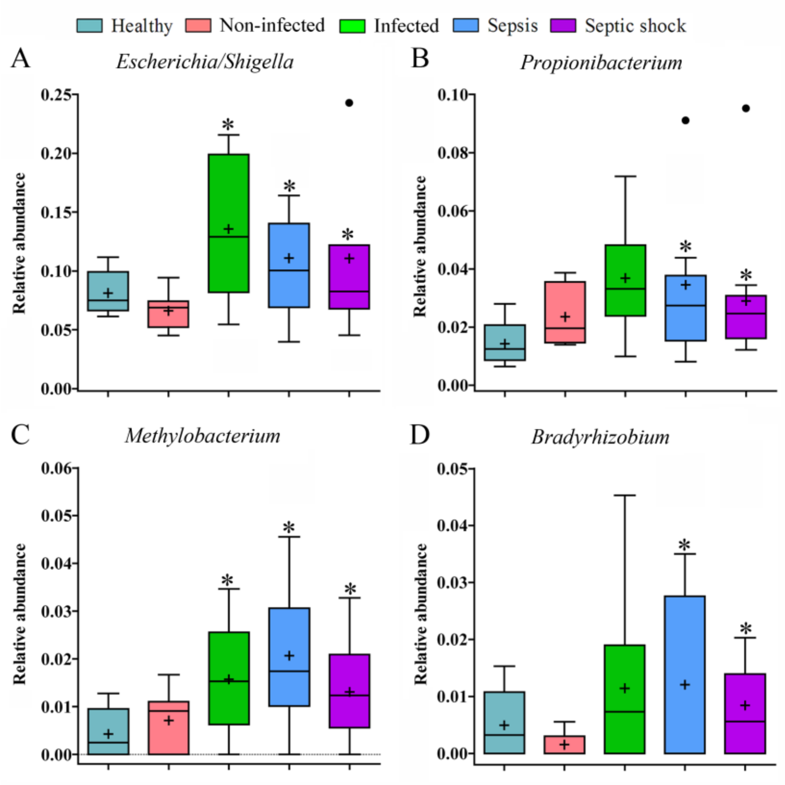

Figure S3. The blood bacterial genera showing significant enriched in septic patients. * *p*<0.05, *vs*. Healthy.

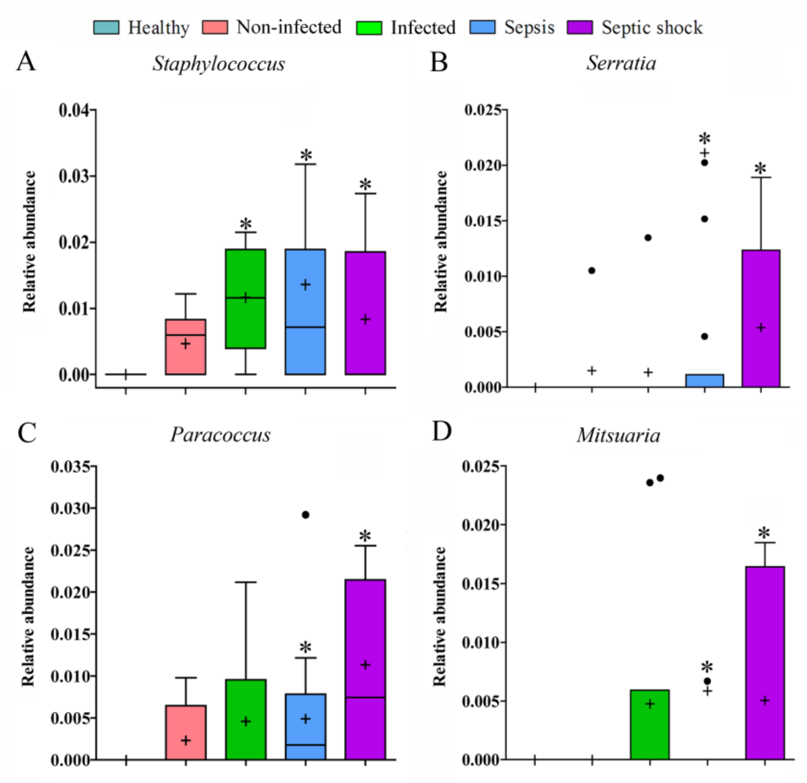

Figure S4. The blood bacterial genera presented in septic patients while absent in healthy individuals. * p<0.05, *vs*. Healthy.

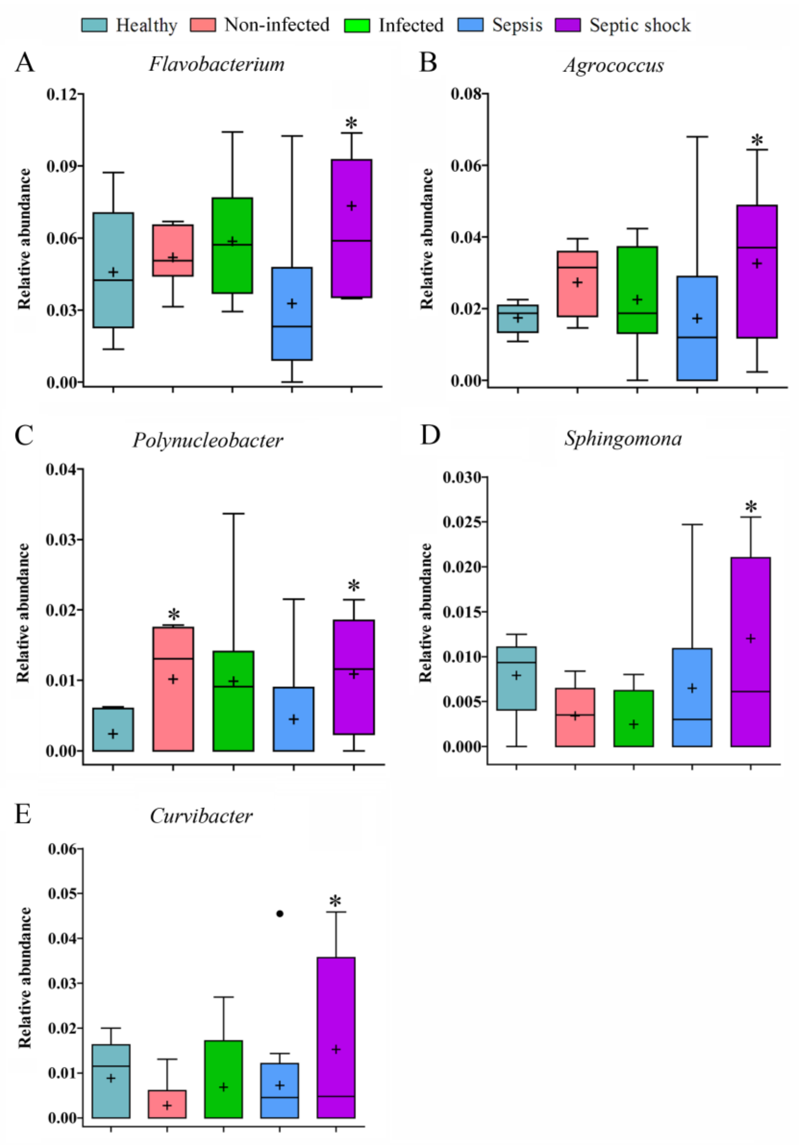

Figure S5. The blood bacterial genera significantly enriched in patients with septic shock. * p<0.05, *vs*. Healthy.

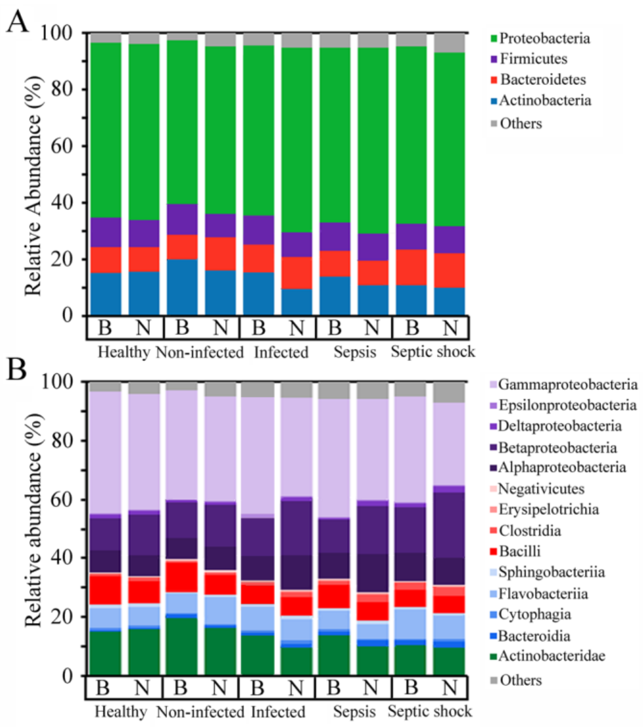

Figure S6. Bacterial composition of the blood and neutrophil-specific microbiotas. (**A**) Comparative analyses at the phylum level. (**B**) Comparative analyses at class level. The letters "B" and "N" represent the blood and neutrophil-specific microbiome, respectively.

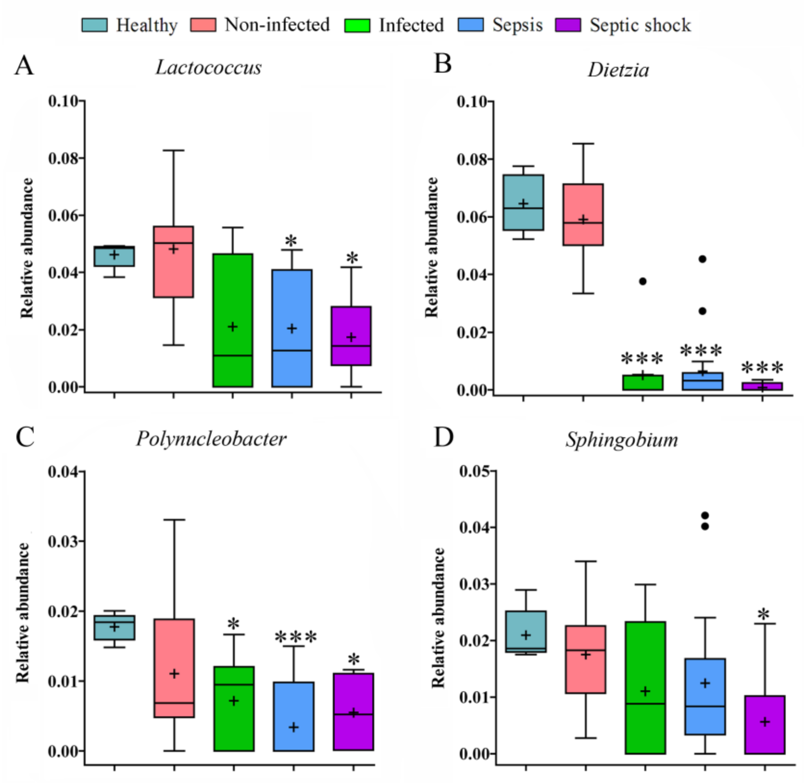

Figure S7. The predominant bacterial genera markedly declined in the neutrophils of septic patients. * *p*<0.05, *** *p*<0.001, *vs*. Healthy.

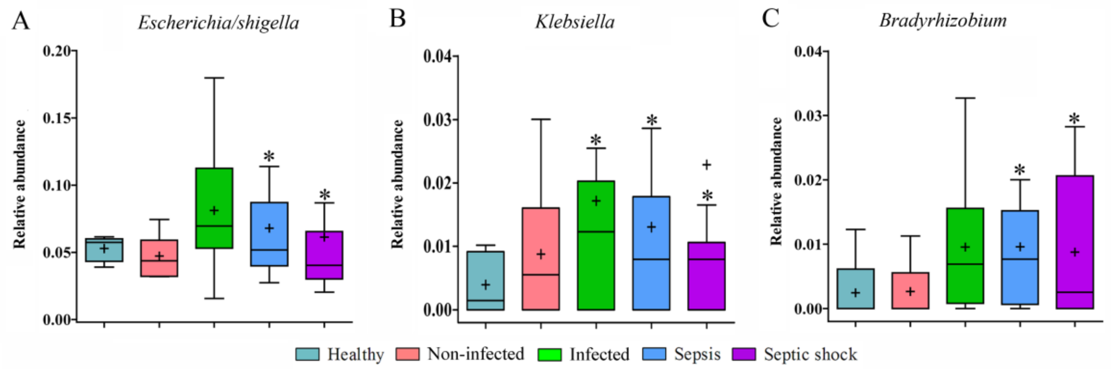

Figure S8. The predominant bacterial genera significantly enriched in the neutrophils of septic patients. * $p<0.05$, *vs*. Healthy.

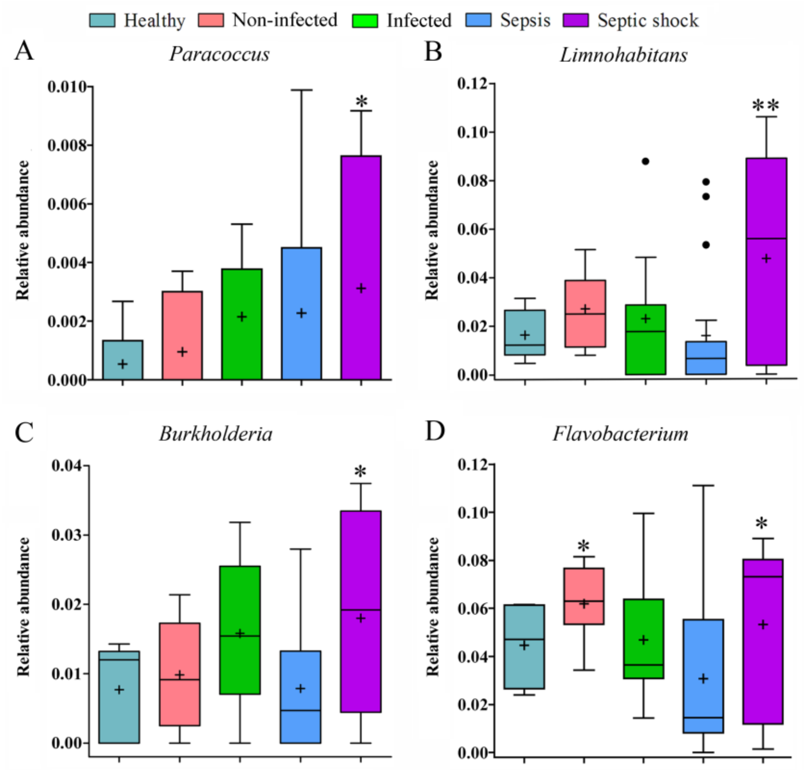

Figure S9. The bacterial genera significantly enriched in the neutrophils of patients with septic shock. * p<0.05, *vs*. Healthy subjects.

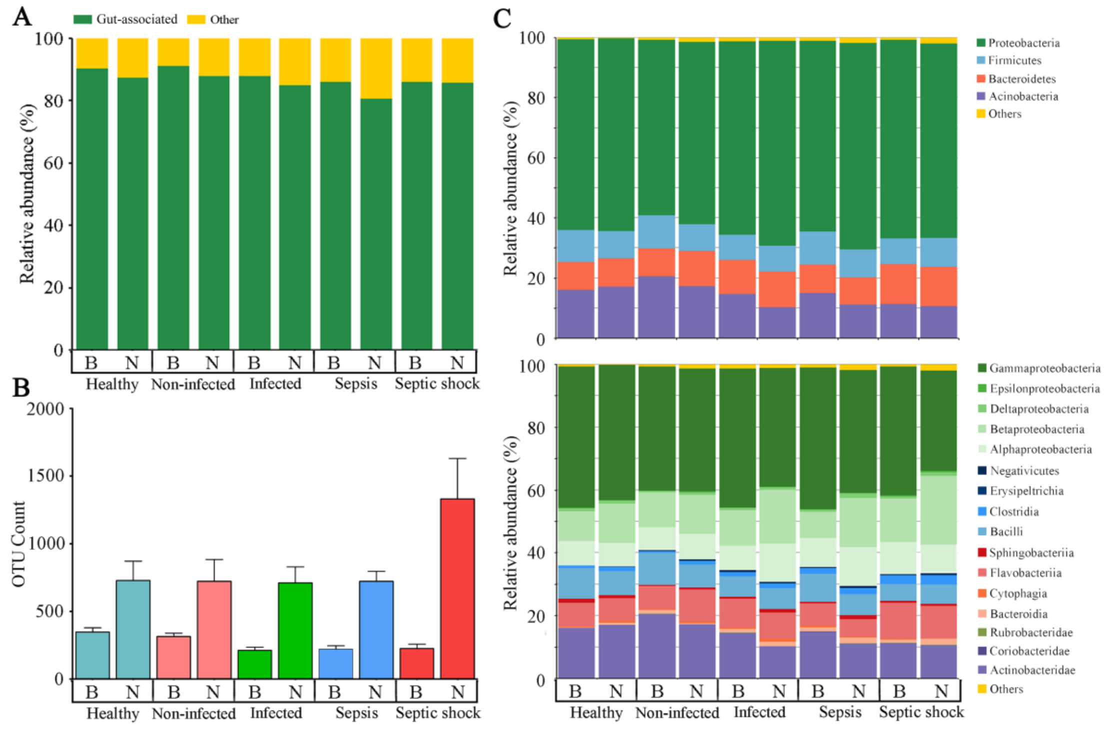

Figure S10. The gut-associated bacterial taxons in the blood and neutrophil-specific microbiomes. **(A)** The relative proportions of the gut-associated bacteria in the blood and neutrophil-specific microbiotas. **(B)** The counts of operational taxonomic units (OTUs) (97% identity) classified into gut-associated bacteria. **(C)** Comparative analyses of the gut-associated bacterial communities at the phylum and class levels. The letters "B" and "N" represent the blood and neutrophil-specific microbiome, respectively.

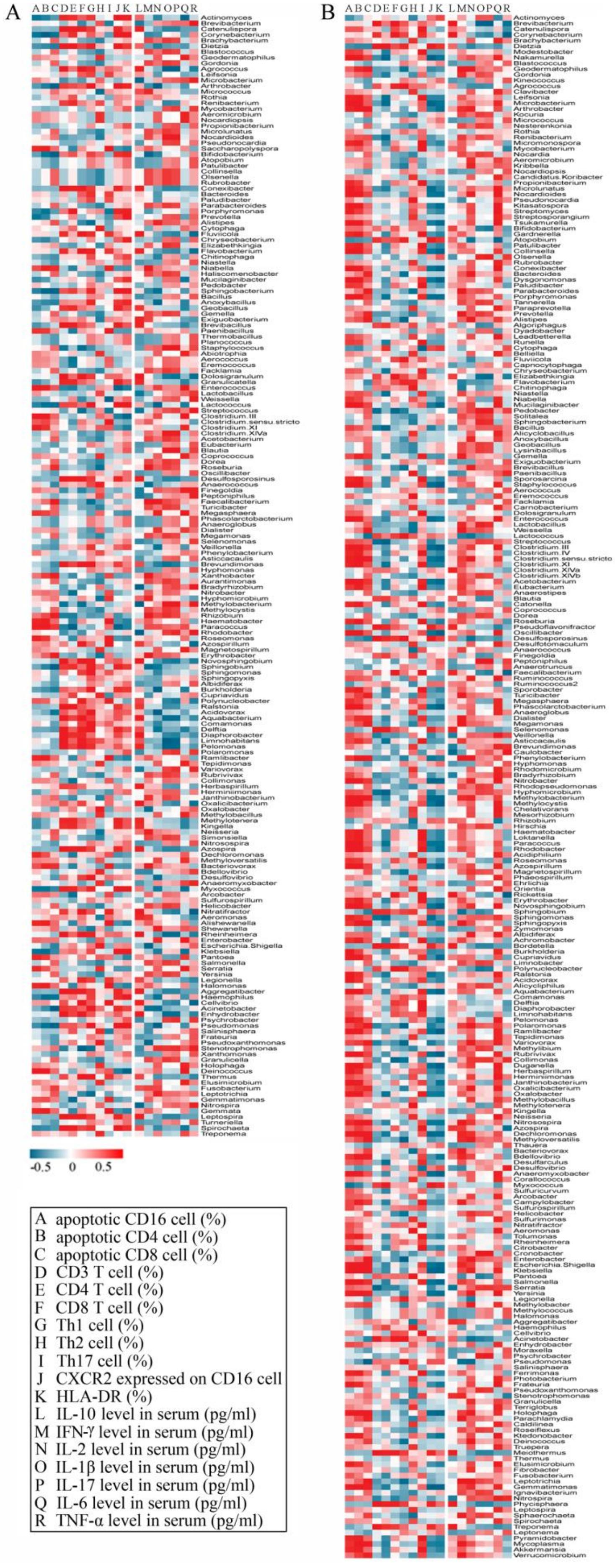

Figure S11. The potential relationship of the immunological changes with the bacterial genera. **(A)** Correlations between the immunological parameters of septic patients and the bacterial genera in blood microbiome. **(B)** Correlations between the immunological parameters of septic patients and the bacterial genera in neutrophil-specific microbiome.
